# The Pivotal Role of Stem Cells in Veterinary Regenerative Medicine and Tissue Engineering

**DOI:** 10.3390/vetsci9110648

**Published:** 2022-11-21

**Authors:** Hussein M. El-Husseiny, Eman A. Mady, Mahmoud A. Y. Helal, Ryou Tanaka

**Affiliations:** 1 Laboratory of Veterinary Surgery, Department of Veterinary Medicine, Faculty of Agriculture, Tokyo University of Agriculture and Technology, Fuchu-shi 183-8509, Tokyo, Japan; 2 Department of Surgery, Anesthesiology, and Radiology, Faculty of Veterinary Medicine, Benha University, Moshtohor, Toukh, Elqaliobiya 13736, Egypt; 3 Laboratory of Veterinary Physiology, Department of Veterinary Medicine, Faculty of Agriculture, Tokyo University of Agriculture and Technology, Fuchu-shi 183-8509, Tokyo, Japan; 4 Department of Animal Hygiene, Behavior and Management, Faculty of Veterinary Medicine, Benha University, Moshtohor, Toukh, Elqaliobiya 13736, Egypt; 5 Department of Animal Medicine, Faculty of Veterinary Medicine, Benha University, Moshtohor, Toukh, Elqaliobiya 13736, Egypt

**Keywords:** stem cells, mesenchymal stem cells, regenerative medicine, tissue engineering

## Abstract

**Simple Summary:**

The employment of stem cells in the treatment of diverse animals’ illnesses and the engineering of various body tissues continues to evolve quickly. Many animal models have been utilized to assess the safety and efficacy of cell-based therapies. They were exploited mainly at a preclinical scale to measure the potential of the application of such therapies in humans and/or animals. Significant research endeavors are conducted to expand their use to include clinical cases. Herein, the main objective of the present article is to discuss the research efforts conducted to use stem cell therapies in veterinary practice, either on experimental animals or clinical cases based on the currently available knowledge.

**Abstract:**

The introduction of new regenerative therapeutic modalities in the veterinary practice has recently picked up a lot of interest. Stem cells are undifferentiated cells with a high capacity to self-renew and develop into tissue cells with specific roles. Hence, they are an effective therapeutic option to ameliorate the ability of the body to repair and engineer damaged tissues. Currently, based on their facile isolation and culture procedures and the absence of ethical concerns with their use, mesenchymal stem cells (MSCs) are the most promising stem cell type for therapeutic applications. They are becoming more and more well-known in veterinary medicine because of their exceptional immunomodulatory capabilities. However, their implementation on the clinical scale is still challenging. These limitations to their use in diverse affections in different animals drive the advancement of these therapies. In the present article, we discuss the ability of MSCs as a potent therapeutic modality for the engineering of different animals’ tissues including the heart, skin, digestive system (mouth, teeth, gastrointestinal tract, and liver), musculoskeletal system (tendons, ligaments, joints, muscles, and nerves), kidneys, respiratory system, and eyes based on the existing knowledge. Moreover, we highlighted the promises of the implementation of MSCs in clinical use in veterinary practice.

## 1. Introduction

The key purpose of tissue engineering is to renovate damaged tissues and replace them with new functional ones. Consequently, these tissues can restore their entire function. This process is multidisciplinary and needs the study of biochemistry, cell biology, developmental biology, biomaterials, and bioengineering [1]. Over the years, a wide range of therapeutic choices have been investigated in different animal models and at diverse scales of research to be used for animals’ and humans’ regenerative therapeutic purposes [2,3,4,5,6,7]. However, the subject of stem cells continues to pique the scientific community’s curiosity. They can be classified as cells with the capacity for self-renewal and cell differentiation in the most basic sense [8,9]. From the time the ovum is fertilized until the time of death, these cells are found in every living thing. Their existence enables the tissues to grow and keep the equilibrium of somatic cells. They also support organ and tissue regeneration by swapping out somatic cells that age or sustain injury [10]. The development of techniques for obtaining stem cells has brought us considerably closer to realizing humanity’s long-held goal of replacing diseased and worn-out cells and/or tissues with fresh ones that are produced in a lab. The Nobel Prize, given to John Gurdon and Shinya Yamanaka for producing what is known as induced pluripotent stem cells (iPSCs), served as a reminder of the significance of stem cells in medicine. They are modified somatic cells that develop stem cell characteristics [11]. It is now possible to employ stem cells in human and animal medicine as a result of countless studies on them in different scientific domains [12,13]. Numerous authors have cited the following characteristics of stem cells: their simple composition and deficient differentiation; self-renewal, which enables maintenance of a persistent population of cells over the life of an animal; asymmetric division, which produces a larger and a smaller stem cell that undergoes additional linear differentiation; the capacity to discriminate into cells of many tissues; and the expression of proteins [8,14,15,16]. The information that is currently known regarding stem cell usage in veterinary medicine is included in this publication. Based on chosen scientific papers, it covers different stem cell types, their immunomodulatory characteristics, and an assessment of the therapy’s efficacy in the treatment of the heart, skin, digestive system (mouth, teeth, gastrointestinal tract, and liver), musculoskeletal system (tendons, ligaments, joints, muscles, and nerves), kidneys, respiratory system, and eyes in the veterinary field.

## 2. Stem Cells Classification

Stem cells are distinguished by being undifferentiated cells present at diverse life stages from the embryonic to the adult stage, producing the adult cells of various body tissues and organs. They are classified based on variable criteria, including their differentiation potential, their origin, and their relationship to the recipient [17].

### 2.1. Differentiation Potential

**Figure 1:**
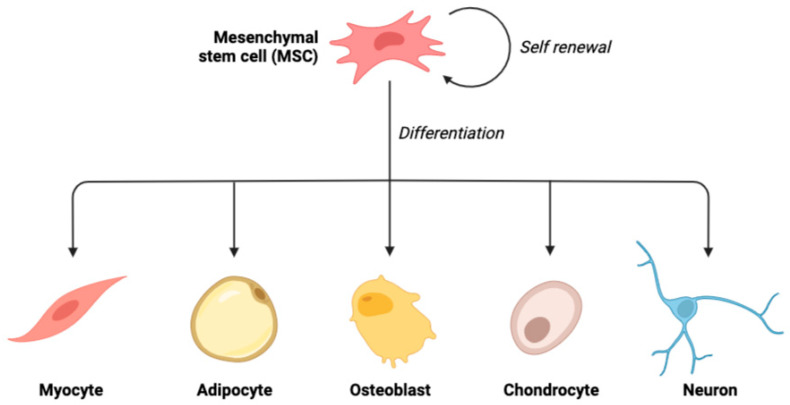
Schematic illustration of the ability of mesenchymal stem cells to undergo multilineage differentiation.

Concerning the differentiation capacity of stem cells, they may be totipotent, pluripotent, multipotent, oligo-/bipotent, or unipotent [17]. Totipotent stem cells are those cells with an infinite capacity for division. Due to their ability to differentiate into any cell that contributes to the development of the embryo and extra-embryonic tissues (placenta, umbilical cord), these cells can give rise to the complete body. As shown by monozygotic twins made from several blastomeres, the totipotent cell is a fertilized ovum (zygote), and cells are acquired from the first germinal stage (morula) [18,19]. Pluripotent stem cells are the cells that have the potential to differentiate into cells that emerge from the germ layers: endoderm, ectoderm, and mesoderm. Hence, unlike the totipotent cells, they are unable to give rise to the full body tissues. The blastocyst germ cells, also known as the inner cell mass (ICM), are an example of pluripotent cells [20]. Multipotent cells are the cells with an outstanding ability to differentiate into various kinds of cells developed from a single germ layer (germ layer-derived cells) [21,22] (Figure 1). They are characterized by their unique proliferation potential, their potential to differentiate into diverse body cells of various origins, and their correlation to the receiver. They can be obtained from diverse tissues such as adipose tissue, bone marrow, umbilical cord blood, and bone Wharton’s jelly. Mesenchymal stem cells are the most investigated multipotent stem cells [17,23]. Oligo-/bipotent stem cells are those cells with the capacity to differentiate into two or more lineages inside a specific tissue. For instance, swine ocular cells have been proven to produce both conjunctival and corneal cells [24]. Unipotent stem cells, also known as precursor cells, exhibit a specialized differentiation pathway into a particular kind of adult body cell. The reproductive layer cells of the epidermis are one example of how they enable the maintenance of a consistent cell population in the tissues under normal circumstances [22].

### 2.2. Origin of Stem Cells

**Figure 2:**
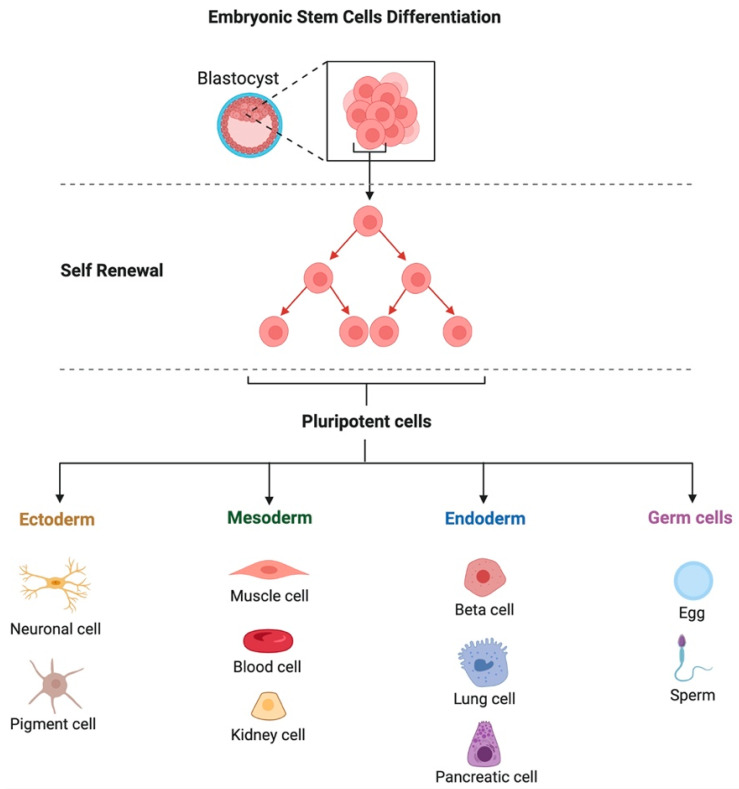
Schematic illustration of the source and multilineage differentiation of embryonic stem cells.

According to their origin, stem cells may be embryonic, adult, tissue-derived, or iPSCs [17]. Totipotent embryonic stem cells (ESCs) are those cells isolated from the embryo’s blastomeres. However, the most often employed pluripotent cells for experimentation are those from the blastocyst embryonic node. Fetal tissues, umbilical cord tissues (such as Wharton’s jelly), umbilical cord blood, and amniotic cells are the sources of fetal stem cells (FSCs) [25]. Embryonic stem cells have an unlimited capacity for self-renewal and differentiation into all cell types *in vitro* (Figure 2). However, it has not yet been feasible to establish exact methods for controlling their differentiation and division at the site of injection *in vivo*, which might, among other factors, result in the emergence of cancerous lesions realized in experimental animals [26]. These challenges, along with ethical concerns, are the major causes of the lack of ESC utilization in clinical stem cell treatment [27]. Somatic or mature cells are synonyms for adult stem cells (ASCs). They are multi- or unipotent, undifferentiated cells.

The possibility of tissues and organs in which they exist is given by their occurrence in the body throughout the postnatal period [25,28]. As an alternative to pluripotent cells of embryonic origin, autologous stem cells of a pluripotent type can be produced by converting adult somatic cells, such as fibroblasts (cultured *in vitro*). They are produced by inserting the genes for the transcription factors (c-Myc, Klf 4, Oct 3/4, Sox 2) required for the formation of embryonic cells. Induced pluripotent stem cells are the resultant cells, which may develop similarly to embryonic cells [27]. Unfortunately, this process is not very successful, and when laboratory mice were given these cells, they developed teratomas such as ESC [10].

### 2.3. Relationship to the Recipient

Based on their relation to the recipient, stem cells can be autogenic, allogeneic, and xenogeneic [29]. Autogenic cells are the most currently investigated. These cells are separated from the recipient animal that is also the donor (for example, bone marrow, adipose tissue, or cord blood). Allogeneic stem cells are harvested from a different animal of the same species. They serve as the cornerstone of therapies that employ both adult and embryonic stem cells [28]. The utilization of allogenic stem cells provides ready-to-use products that enable suitable timing of the regenerative therapy implementation and skip the required time for the expansion of the cultured autogenic cells. However, the key restriction factor of this cell type is the need for a donor. Xenogeneic stem cells are alternatives to autogenic and allogenic stem cells [30]. Xenogenic stem cells can be harvested and obtained from various animals of different species. Numerous investigations using antlerogenic (xenogenic) cells revealed that some species, including rabbits and horses [31], had a regenerative response when their tissues were exposed to stem cells denoted by the macrophage inhibitory cytokine 1 (MIC-1) [32].

## 3. Sources of MSCs in Tissue

Mesenchymal stem cells may be effectively extracted from practically any tissue in a live organism. The finest outcomes are, however, attained when extracting cells from adult tissues such as adipose tissue (AD-MSCs), bone marrow (BM-MSCs), peripheral blood (PB-MSCs), or fetal tissue such as the placenta (P-MSCs), umbilical cord blood (UCB-MSCs), or umbilical cord (UC-MSCs) [33,34]. This is because of the proliferative and differentiation potential of these cells. It was discovered that the kind of cells, based on their origin, has a substantial influence on both their ability to differentiate *in vivo* and their physiologically important characteristics [26]. The usage of MSCs in a particular therapy should be a key consideration when choosing the source of those cells. Bone marrow is one of the most researched sources of MSC origin in veterinary treatment, similar to human medicine [35]. Sadly, collecting the samples is an intrusive operation done under the effect of general anesthetic in canines and sedatives with or without local anesthetic in equines, both of which have a danger of postoperative problems, including infection and/or bleeding [36]. Several reports of serious unintentional cardio-thoracic punctures [37] as well as nonfatal pneumopericardium in horses [38] have been published. However, in these animals, the BM-MSC collection process may be carried out on a standing animal with little to no danger of the previously stated problems. Today, it is acknowledged that sternal biopsy utilizing a Jamshidi needle at the 4th or 5th sternebra is the most appropriate location [39,40]. The quantity of BM-MSCs diminishes with aging and makes up a very minor portion of all bone marrow stromal mononuclear cells [41]. The aspirate that was previously taken from the donor is used for MSC culture and passage *in vitro*. The therapeutic number of MSCs strictly relies on the number of cultured MSCs, which can be passaged four times maximum. This culture includes various phases (establishing of the culture, cell growth, change of the medium, and completion of the culture with the determination of the cell’s phenotype).

Adipose tissue has grown in popularity and accessibility as a source of material for MSC extraction and separation in recent years [42]. The simplest method of collecting the material is a planned lipectomy or lipoaspiration carried out during preventive ovariohysterectomy in dogs and cats. Subcutaneous or visceral fat has been proposed as a source of AD-MSCs [43]. Immediately upon collection, a small number of nucleated cells with evidence of AD-MSCs may be extracted from the aspirate. Similar to BM-MSCs, it is essential to culture fat-derived cells *in vitro* to acquire a therapeutic dosage. This requires particular laboratory techniques, such as enzymatic digestion, followed by serial washing and centrifugation. Since AD-MSCs have been demonstrated to present a high ability for differentiation as well as proliferation, regenerative medicine routinely uses these cells as a source. These cells have been and are often employed in the therapy of musculoskeletal illnesses, and when compared to BM-MSCs, they have a higher bioavailability and are easier to get [44,45]. An alternate source of MSCs is thought to be PB-MSCs. The least contentious and least dangerous one that does not need pharmacologically sedating the animal is collecting a blood sample, which is superior to the other ways mentioned above. Attributed to the limited bioavailability of PB-MSCs in the peripheral blood of dogs, horses, and humans, the current studies must be continued. Additionally, these cells were effectively extracted from guinea pigs, mice, and rabbits [46]. According to the authors, only 3 out of 10 test horses had adequate blood cells for further cell culture. To boost the quantity of PB-MSCs in the peripheral circulation, these animals were additionally given particular treatments (hyperbaric chambers) [47,48]. Fetal tissues, including the placenta, fetal membranes, amniotic fluid, Wharton jelly (WJ) of the umbilical cord, and umbilical cord blood, are another potential and increasingly often employed source of stem cells in veterinary medicine [49,50]. It is well recognized that MSC performance decreases with donor aging. The ability of cells isolated from fetal tissues to differentiate into not just mesenchymal cells but also cells from the other three germ layers may be a sign of their pluripotency [51]. The process for gathering the material is categorized as a minimally invasive approach and takes place in the perinatal period as opposed to the collection of BM-MSCs or AD-MSCs. The reduced sterility of the method itself may be the noted drawback of the material packing approach [52].

## 4. Autogenous Allogeneous, and Xenogenic MSCs

**Figure 3:**
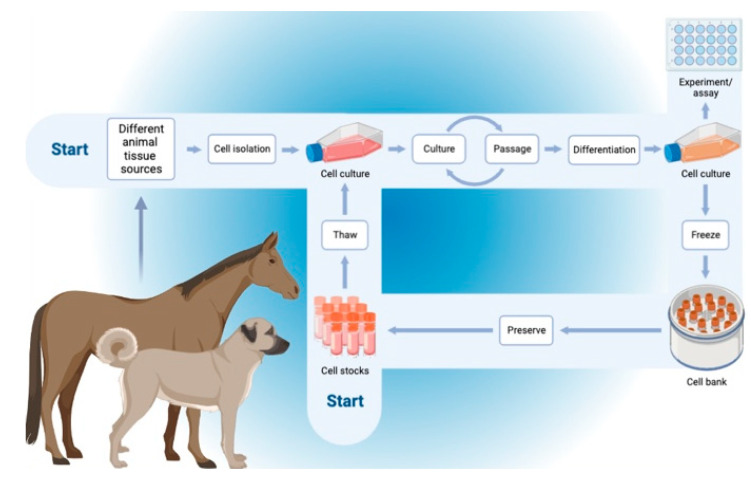
Schematic representation of the workflow of the autogenic and allogenic mesenchymal stem cells from the harvesting, culturing, storage, and use.

Allogeneic and autologous stem cells are the two main kinds of stem cells employed in modern regenerative therapy. The difference in the safety and effectiveness of employing these cells in treatment is a topic of continuous discussion among experts worldwide. As previously indicated, MSCs can be categorized as being of allogeneic, autologous, or xenogeneic origin, depending on the donor-recipient relationship. The recipient of allogeneic cells is a member of the same species as the donor. Compared to autologous cells, successful *in vitro* cultivation of these cells drastically decreases the time needed to perform the therapy. The antlerogenic stem cells MIC-1 (obtained from a deer) were therapeutically administered to horses and experimentally injected into the tissues of injured rabbits [53]. They are the least often employed source of MSCs because the donor and recipient are from different species. Researchers have been attempting to establish whether allogeneic or autologous cells are superior throughout the heyday of regenerative medicine and at the height of the hunt for alternative therapies. One must weigh the benefits and drawbacks of each type of MSC before choosing the kind of therapy to be utilized. The safest source of MSCs, according to logic, should be cells extracted directly from the same animal. Hence, autologous cells are thought to be safer for therapy, but on the other hand, acquiring and preparing a therapeutic amount of these MSCs is a time-consuming process that frequently involves surgery during harvesting with high possibilities of infection and disease transmission [54]. This technique is only sometimes employed because of the aforementioned issues and the overall expense of the process. The animal’s age, sex, and kind of sickness are all strongly correlated with the quantity and quality of autologous cells in that animal [55,56,57]. On the other side, after donation, allogeneic cells can be cultivated and stored in a cell bank forever (Figure 3). This makes it possible to identify a single-cell batch with a stable MSC quantity, safety level, and potential for differentiation.

Such a strategy would shorten the time required for material collection and cell growth, minimize usual variability, as in the autogenic cells, and allow for uniformity of the therapy and the anticipated outcomes [57,58]. The national legal guidelines about the utilization of medical preparations should also be considered when using allogeneic cells. Bertoni et al. [59] provided intriguing test findings after analyzing the impact of auto- and allogeneic stem cells on the fetlocks of healthy horses. Although there was no discernible change in the characteristics of the synovial fluid, it was declared that BM-MSCs considerably increased synovial effusion when compared to UCB-MSCs. Compared to the placebo, MSC injections caused slight to moderate topical inflammatory symptoms, whereas more cells showed reduced inflammation during clinical and ultrasonographic tests [59]. Two allogeneic cell treatment products for horses and one for dogs that have a marketing license from the European Medicines Agency, among others, are proof of the tolerance of allogeneic stem cells [60]. Many investigations have studied the use of xenogeneic stem cells. Various experimental animals, including mice, rats, rabbits, dogs, and baboons, were used to cultivate these cells both locally and systemically [61]. Studies on rats revealed that 12 weeks after being injected into the carotid artery, xenogeneic cells (murine) were present in the bone marrow. In addition to the bone marrow, their presence has been detected in the myocardial infarction tissue as immature myocytes [62]. Further effective trials to use xenogeneic cells have been reported. For instance, rat stem cells were used to regenerate bone tissue in rabbits [63], human bone marrow stem cells were used to treat rat spinal cord injuries [64], and the same cells were used to regenerate bone in mice [65]. A growing number of researchers believe that the use of xenogeneic stem cells can replace the use of allogeneic or autogenous stem cells in regenerative medicine after analyzing the already available data. The immunogenicity of xenogeneic stem cells is comparable to that of autologous and allogeneic stem cells, according to studies [25,28].

## 5. Immunomodulatory Potential of MSCs

The presence of the major histocompatibility complex (MHC) on the surfaces of the living cells plays a key role in the detection of the acceptance or rejection of the donated tissue by the recipient’s body. Hence, there is a possibility for the rejection of the allogenic cells or organs. Almost every nucleated cell in the body possesses a class I MHC (MHC I) molecule, whereas class II molecules (MHC II) are associated with immune cells such as macrophages, dendritic cells, and lymphocytes. As a result, recipient CD8+ T cells detect every allogeneic cell with MHC I surface molecules, resulting in the direct cytotoxicity of the foreign cells. Moreover, they can be identified by the recipient CD4+ T cells if they carry MHC II molecules, which can trigger a cytotoxic or humoral immune response. After being indirectly recognized by antigen-presenting cells, B cells may also produce alloantibodies [66]. Despite the absence of clear information on their immunogenicity, adult MSCs are thought to be low-immunogenic. This is attributed, in part, to the lack of MHC II antigens on their surface and the poor expression of the MHC I. They prevent NK cells, T lymphocytes, and B lymphocytes from proliferating or functioning normally. Additionally, they block antigen-presenting cells and promote the growth of suppressor T cells [67]. Only MHC I molecules are expressed on the surface of MSCs following their stimulation to differentiate along the adipogenic, osteogenic, and chondrogenic lineages [68]. The allogeneic variety of MSCs may be a widely available source for regenerative therapy in veterinary medicine since they appear to be immunologically privileged. However, it is important to consider the possibility that the receiver may contract the donor’s illness [69]. Hence, it is necessary to schedule a specialized systemic strategy for the management of possible viral, bacterial, and fungal contaminations for each species. The MSC secretome is rich in anti-inflammatory cytokines such as interleukin 18 (IL18), IL13, IL10, neurotrophin 3 (NT-3), and ciliary neurotrophic factor (CNTF). Thus, it presents an outstanding immunomodulatory ability [70]. Furthermore, it has been proven to be safer than MSCs despite being administered in high doses in some cases without major adverse effects. Additionally, its use exhibits a lower possibility of emboli formation post-intravenous administration or pathological or tumorigenic transformations compared to those caused by the hysterical cell differentiation. In addition, it allows the utilization of allogenic therapies without immune stimulation as it represents the structure of the parental cells [71].

## 6. Clinical Applications of Stem Cells in Regeneration and Bioengineering of Different Body Organs and Tissues in Veterinary Practice

Stem cells have so far been employed, primarily at experimental scales, to cure a wide range of illnesses in many animal species. The early focus of veterinary regenerative medicine was on orthopedic illnesses, but it is currently swiftly spreading to include additional conditions, such as those affecting the heart, digestive tract, liver, kidneys, lungs, nerves, and muscles. Stem cells have frequently been employed in dogs and horses with various tissue diseases. Moreover, in cats, renal and respiratory affections and several inflammatory disorders were treated with these cells [26]. In this section, the employment of stem cell therapy for engineering and treating of different body tissue affections in diverse animals is discussed, and some representative examples are presented in Table 1.

### 6.1. Heart

Cardiovascular diseases are the leading reason for death in humans worldwide. Likewise, they are very serious in animals as well, with the challenge that most cases passed go undiagnosed. However, the introduction of novel cardiac imaging tools in veterinary medicine has significantly helped in the early diagnosis or even prediction of cardiac dysfunctions [72,73,74,75,76,77,78,79,80,81]. That, in turn, will help to refer them to available therapeutic options.

**Table 1:** Employment of diverse kinds of stem cells in the regeneration of various body tissues in different animals.

Body Systems and Tissues		The Type of Cells Used	Animal Model	Route of Administration	Outcomes	Refs.
**Heart**		Adipose-derived mesenchymal stem cells (AD-MSCs)	Dobermann dogs with dilated cardiomyopathy	Retrograde coronary venous delivery	The stem cell therapy was safe. However, it did not prevent the progression of the disease	[82]
		Allogenic Cardiosphere-derived cells (CDCs)	Doberman pinscher dogs with spontaneous DCM	Intracoronary infusion	Safety was confirmed with an effective improvement of the heart functions	[83]
		Allogenic puppy deciduous teeth stem cells (PDSCs)	Dogs with chronic valvular heart disease	Intravenous injection	Amelioration of the cardiac functions and improvement of the quality of the life scores	[84]
**Skin**		Caprine amniotic fluid and bone marrow-derived mesenchymal stem cells	New Zealand white rabbits	Subcutaneous injection	Amniotic fluid-derived cells were superior to bone marrow-derived cells for enhancement of skin wound healing	[85]
		Peripheral blood-derived MSCs	Sheep	Subcutaneous injection in the margins of the skin wound	Improvement of both superficial and deep wound healing	[86]
		Umbilical cord-blood-derived equine MSCs	Horses	Subcutaneous injection in the margins of the skin wound	Stem cell therapy is a promising choice for the treatment of distal extremity wounds in horses	[87]
**Digestive System**	**Mouth and teeth**	Adipose-derived multi-lineage progenitor cells (ADMPC)	Micro-mini pig	Topical transplantation of autologous or allogeneic ADMPC-fibrin gel complex	ADMPC presented an immune-modulation action and enhanced periodontal tissue regeneration	[88]
		Dental pulp stem cells (DPSCs)	Mongrel dogs	Direct pulp capping method	DPSCs have exhibited a promising capacity for regeneration of the damaged dentin.	[89]
		Mobilized dental pulp stem cells (MDPSCs) isolated from the abdominal subcutaneous adipose tissue	Dogs	Topical transplantation in the pulpectomized dogs	Safety was confirmed with the enhancement of total pulp regeneration	[90]
	**Gastrointestinal tract**	Adipose tissue-derived MSCs (AD-MSCs)	Dogs with inflammatory bowel disease (IBD)	Intravascular (IV) infusion	IV infusion of AD-MSCs was safe and effective in dogs with IBD	[91]
		Adipose-derived feline mesenchymal stem cells (fMSC)	Cats with chronic enteropathy	Intravenous injection	fMSCs were safe and effective for the treatment of cats with chronic enteropathy	[92]
	**Liver**	Canine adipose-derived mesenchymal stem cells (cADSCs)	Dogs with induced acute liver injury	Intraperitoneal injection	cADSCs played an important role in the regeneration of canine liver	[93]
		Bone marrow-derived mesenchymal stem cells (BM-MSCs)	Dogs with induced liver fibrosis	Intravenous infusion	Improvement of liver functions with no adverse effects	[94]
**Musculoskeletal system**	**Tendons and ligament**	Bone marrow-derived mesenchymal stem cells (BM-MSCs)	Horses with tendon or ligament injuries	Topical intralesional injection	BM-MSCs were safe and successfully regenerated equine tendons and ligaments	[95]
		Bone marrow-derived mesenchymal stem cells (BM-MSCs)	Polo with an injured superficial digital flexor tendon	Topical injection at the lesion site	Improvement of the regeneration capacity of the injured tendon	[96]
	**Joints**	Bone marrow-derived mesenchymal stem cells (BM-MSCs)	Horses with a stifle injury	Intra-articular administration	Improvement of the stifle injury with enhanced ability of the animals to return to work	[97]
		Adipose-derived MSCs (AD-MSCs)	Horses with osteoarthritis	Intra-articular administration	AD-MSCs were efficient and safe	[98]
	**Muscles and nerves**	Neurogenically-induced bone marrow-derived mesenchymal stem cells (NIBM-MSCs)	Dogs with paraplegia	Percutaneous transplantation	NIBM-MSC therapy was a promising option for the treatment of spinal cord injuries	[99]
		Bone marrow-derived mesenchymal stem cells (BM-MSCs)	Dogs with paraplegia	Intralesional injection	Enhanced regeneration of the injured spinal cord	[100]
**Kidneys**		Bone marrow-derived or adipose tissue-derived MSCs (BM-MSCs or AD-MSCs)	Cats with chronic kidney disease (CKD)	Ultrasound-guided intrarenal injection	Improved kidney regeneration and function	[101]
		Amniotic membrane-derived MSCs (AMSCs)	Cats with chronic kidney disease (CKD)	Ultrasound-guided intrarenal injection and intravenous infusion	AMSCs exhibited a renoprotective effect and enhanced kidney function	[102]
**Respiratory system**		Bone marrow-derived mononuclear cells (BMMCs)	Horses with recurrent airway obstruction (RAO)	Intratracheal instillation	BMMCs could reduce inflammatory reactions	[103]
		Human umbilical cord-derived mesenchymal stem cells (MSCs)	Dogs with radiation-induced lung injury	Intratracheal transplantation	Reduced lung injury and declined inflammation	[104]
**Eye**		Bone marrow-derived mesenchymal stem cells (BM-MSCs)	Horses with unilateral immune-mediated keratitis (IMMK)	Subconjunctival injection	Improved corneal clarity with improved regeneration of the corneal tissue	[105]
		Feline adipose-derived mesenchymal stromal cells (fAd-MSCs)	Cats with feline eosinophilic keratitis (FEK)	Subconjunctival injection	fAd-MSCs were safe and effective for the treatment of FEK	[106]

Cardiac stem cell treatments were employed many years ago in human medicine to heal the damaged myocardium after an acute or chronic myocardial infarction [107]. Companion animals seldom get primary myocardial infarction [108]. However, dilated cardiomyopathy is a rather prevalent condition in large dog breeds. This condition will inevitably progress, which will result in refractory congestive heart failure and death. Retrograde coronary venous allogeneic AD-MSCs administration was used in Dobermans as an experimental therapy for this condition. Although safe, stem cell therapy did not present any positive outcomes [82]. Likewise, transplanting allogeneic cardiosphere-derived stem cells into coronary arteries did not improve the condition of animals with dilated cardiomyopathy [83]. The most prevalent cardiac condition in smaller dog breeds is degenerative valve disease, which is frequently complicated by ventricular enlargement and cardiac dysfunction [109]. Petchdee and Sompeewong [84] have investigated the utility of puppy deciduous teeth-derived stem cells administered intravenously in the treatment of degenerative valvular conditions. Even though this was a small trial, the left ventricular ejection fraction improved. Hence, further research will be required to confirm any possible beneficial findings.

### 6.2. Skin

The process of successful wound healing is usually disrupted by inadequate cellular and molecular pathways. This frequently results in a persistent, chronic wound that makes the animal uncomfortable. Owing to their anti-inflammatory and regenerative capabilities, MSCs may thus be a potentially effective therapy for chronic wounds with significant inflammation and a hyperplastic response [110]. The positive effects of MSC therapy on wound healing in caprines [85], ovines [86], horses [87], and canines have been demonstrated in several investigations using animal models [111]. The utilization of MSC-generated extracellular vesicles (ECVs) injected locally to treat dogs’ wounds led to noticeably better cutaneous wound healing [112]. In another work, peripheral blood stem cells (PBSCs) were infused topically and systemically into four horses with spontaneous infected wounds that had not responded to traditional treatments. Within four weeks of therapy, tissue expansion was seen in all four cases as a result of the formation of crusts and tiny scars in the middle of the incision [113]. In a further investigation, full healing of the non-healing skin lesion was also shown in a filly following repeated topical applications of heterologous WJ-generated MSCs with the utilization of carboxymethylcellulose gel. In 5 days, the wound had entirely healed [114]. On the other hand, MSCs were utilized to treat atopic dermatitis, one of the most prevalent skin conditions in dogs, in addition to wound healing. Two trials employing a comparable amount of IV-administered AD-MSCs in dogs with atopic dermatitis have found conflicting outcomes. At first, there was no discernible improvement in the clinical symptoms or pruritus [115]. Twenty-two dogs with atopic dermatitis (AD) who had not responded to conventional treatment were enrolled in the second study. After receiving allogeneic AD-MSCs intravenously for one month, cadesi-04 scores and pruritus were both drastically reduced. Clinical symptoms were in remission for at least 6 months with no side effects noticed [116]. Stem cells may be an intriguing potential therapeutic to support chronic wound healing, according to diverse research conducted on both laboratory and clinical veterinary animals. However, many issues must be resolved in further research before such medicines are used widely in clinical practice. Since there is a dearth of information on AD in dogs, it is hard to foresee, at this time, whether stem cell therapies will be effective.

### 6.3. Digestive System

#### 6.3.1. Mouth and Teeth

The quality of the animal’s life might be significantly impacted by oral discomfort and mastication issues [117]. Recently, oral and dental disorders have become widely prevalent among dogs and cats worldwide and are associated with severe pain and potential topical and systemic infections [118,119]. Stem cells have gained attention for the repair of oro-dental tissues as regenerative cell treatment has advanced. Studies emphasize the ability of MSCs to modulate the immune responses to promote the regeneration of periodontal and dental tissues, as well as their differentiation capability to enhance the strength of the implant and bone tissue healing in alveolar defects. In the mini-pig periodontal defect model, allogeneic AD-MSCs alone could also stimulate the regeneration of periodontal tissue [88]. Autologous [89] and allogeneic [90] dental pulp stem cells, or autogenous BM-MSCs [120], were effective in regenerating canine dental pulp. Even while these studies suggest that oro-dental tissue regeneration is possible, other research suggests that stem cells have no positive effects on dental disorders such as problems with dental implants [121]. Animal model studies do provide a base and a point of reference for the utilization of stem cells and tissue engineering in boosting oro-dental tissue regeneration. The effectiveness and utility of stem cell therapies for the treatment of oro-dental issues in animals with natural disorders, however, still need significant investigation. Feline chronic gingivostomatitis (FCGS) is a painful and crippling oral illness in cats characterized by persistent gingival inflammation that extends to the buccal and caudal oral mucosa. Anorexia, oral discomfort, weight loss, ptyalism, halitosis, and a lack of grooming are the major symptoms of FCGS [122]. Currently available treatments include corticosteroids [123], cyclosporin [124], and tooth extraction surgery [125], and have varying response rates and many potential side effects [126]. The employment of MSC therapy in FCGS has shown extremely positive results. In most cats, full clinical and histological remission or a decrease in the severity of the clinical condition were the outcomes of IV therapy with autogenic AD-MSCs, according to research by Arzi et al. [127]. The stabilization of immune cell subsets, cytokine levels, and serum protein levels served as proof of the immunomodulation of MSCs. The study’s findings also indicated that the lack of cells with low expression of CD8 (CD8lo cells) could serve as a biomarker to gauge how well MSC treatment will work. It is interesting to note that allogeneic AD-MSCs appear to have poorer clinical effectiveness than autogenic MSCs in the treatment of FCGS [128]. About 70% of cats with FCGS treated with IV allogenous AD-MSC had a clinical, histopathological, and systemic response [29].

#### 6.3.2. Gastrointestinal Tract

Inflammatory bowel disease (IBD) is an idiopathic disorder of the gastrointestinal tract distinguished by continual or repeated gastroenteric symptoms with inflammatory evidence with unknown underlying reasons [129,130]. Some dogs are resistant to the standard, ongoing therapies, including cyclosporine or steroids. Nine out of 11 dogs with severe IBD experienced clinical remission following a single IV infusion of allogenic AD-MSCs six weeks after the treatment, and blood levels of albumin, cobalamin, and folate significantly increased [91]. In cats, IBD is also rather typical, with frequent vomiting and diarrhea. Allogeneic AD-MSCs were used to treat cats with IBD in placebo-controlled, blinded research. Five out of seven cats’ owners reported considerable improvement or full remission of the clinical symptoms. In contrast, there was no improvement in the clinical signs or perhaps deterioration in cats who received the placebo [92]. Attributed to their immunomodulatory and anti-inflammatory properties, MSCs appeared to be a potential alternative therapy for canines and felines with IBD. The preliminary study findings are encouraging, but larger follow-up studies and more investigation are required to prove that MSC therapy is a secure and efficient means of treating IBD in animals.

#### 6.3.3. Liver

Stem cell therapies for canine liver disease were the subject of several investigations. In an investigation, Yan et al. [93] explored the impact of IV injection of autogenic AD-MSCs for the treatment of experimentally induced canine acute hepatic damage. The homing of AD-MSCs to the liver decreased the blood-liver enzyme levels, and the restoration of hepatic tissue structure following the therapy points to the possibility of using MSCs to treat liver disorders in canines. Additionally, MSCs were used in a canine liver cirrhosis model. Without causing any negative side effects, IV administration of autogenic BM-MSCs dramatically reduced the amount of hepatic fibrosis and enhanced hepatic function in the cell received group [94]. In a canine model of liver fibrosis, IA administration of BM-MSCs was demonstrated to be safe, similar to IV administration, but intriguingly, IA application of MSC had a longer-lasting impact on lowering liver enzyme levels in the peripheral blood [131]. Additionally, 10 dogs with degenerative hepatopathy were repeatedly treated with autogenic AD-MSCs intravenously. When compared to the control group, all animals showed noticeably better liver function in terms of the drop in hepatic biomarkers following each treatment [132]. Additionally presented was a clinical instance of MSCs-treated hepato-cutaneous syndrome. Allogeneic AD-MSCs were repeatedly injected intravenously or into the hepatic parenchyma. For this condition, the dog’s survival with relapsed or limited clinical indications was longer than anticipated [133]. The IV route of delivery seems reasonable for treating hepatic disorders that are sensitive to the MSC treatment in animals because IV injection of MSCs causes the buildup of cells in the liver after they have been removed from the lungs [134]. The few trials that have been done on clinical cases make it difficult to draw firm conclusions on the optimum delivery method as well as the effectiveness and safety of allogenic MSCs in treating hepatic disorders. Therefore, further research is required to solve these difficulties.

### 6.4. Musculoskeletal System

#### 6.4.1. Tendons and Ligaments

Tendon and ligament traumatic and stress injuries naturally recover with the growth of scar tissue, which is less functional than healthy tissue. While the initial damage reduces structural stiffness, fibrosis destroys the tendon or ligament’s physiological architecture and function [135]. This leads to impaired locomotor function that is vulnerable to further harm [135]. Therefore, the goal of the ideal treatment should be to restore to the tissue’s natural structure and function. Traditional treatments for tendon injuries in horses include chilling [136], bandaging, and a period of recovery during which regulated activity is performed. Corticosteroids or other anti-inflammatory medications can be used as pharmacological therapy; however, surgery is frequently necessary [137,138]. Reinjury is frequent with these conservative methods, and animals are frequently not able to function as well as they did before the injury [137]. Therefore, the ideal course of treatment should focus on tendon matrix regeneration. Due to its potential as a tool for improved tissue regeneration, the use of MSCs has been proposed as a substitute for the conventional strategy [95]. The goal of regenerative cell-based treatment is healing with correct collagen fiber production and effective restoration of normal tendon function with a reduced chance of recurrence. It is anticipated that the most suitable source of MSCs for stem cell therapy will be MSCs derived from the same tissue that needs to be treated. Therefore, tendon-derived stem cells would be the ideal source of stem cells for tendinopathies [139], but it is exceedingly difficult to isolate stem cells from tendonous tissues, and there is no accepted stimulation procedure for tendon genesis [140]. Therefore, for tendon regeneration, stem cells from several sources, primarily from adipose tissue and bone marrow were employed. First described in 2003, autogenic BM-MSC transplantation into the equine superficial digital flexor tendon [96]. Significant clinical healing was documented when cells were injected into 11 racehorses with superficial digital flexor tendon injuries [141]. Similarly, intralesional administration of autogenous BM-MSCs caused 28% or more reinjuries in all 141 racehorses included in the cohort research with naturally occurring superficial digital flexor tendon injury [35]. When horses were given intralesional injections of beta aminopropionitrile fumarate, hyaluronan, or polysulfated glycosaminoglycans for the same kind of damage and follow-up, the results demonstrated a considerable decrease in the rate of reinjury [142]. Smith et al. [143] revealed that autogenic BM-MSC therapy of natural tendinopathies causes the production of tissue matching a typical tendon matrix as opposed to the fibrous tissue that is generated throughout the normal healing procedures. Besides autogenous MSC therapy, allogeneic MSC therapy has shown promise in treating tendon and ligament problems such as desmitis of the suspensory and inferior check ligaments and tendinitis of the superficial and deep digital flexor tendons [144]. However, as compared to alternative therapies such as platelet-rich plasma (PRP), autogenic BM- or AD-MSC therapy showed little or extremely little enhancement in surgically produced lesions of the horse’s superficial digital flexor tendons [145,146]. Dogs were also given experimental MSC therapies, much the same as horses. A cranial cruciate ligament rupture in the stifle joint is a frequent injury in dogs [147]. Its rupture is the most characteristic cause of lameness in older dogs and is connected to stifle osteoarthritis [148]. Currently, surgical correction is the advised course of treatment [149]. The efficacy of MSC usage in this disease was underlined by positive treatment outcomes from multiple investigations. It has been shown that dogs needing tibial plateau leveling osteotomy (TPLO) may experience less post-operative pain and lameness following a single intra-articular injection of allogenic BM-MSCs than they would following a one-month course of oral non-steroidal anti-inflammatory drug (NSAID) administration [150]. According to research [151], autogenic BM-MSCs administered intraarticularly engraft in the region of damaged cranial essential ligaments and have an anti-inflammatory effect. In dogs with the same disease, post-operative intraarticular or IV injection of autogenic MSCs led to lower CD8+ T-cell counts, serum and synovial CRP, and synovial IFN- levels that lasted for more than 8 weeks after BM-MSC injection [152]. Promising findings were gathered from the retrospective study, where autogenous BM-MSC treatment combined with PRP avoided additional degeneration of the joint and canine contralateral ligament rupture in cases of incomplete tears without disruption of the stifle joint, where surgery is not the best treatment [153].

#### 6.4.2. Joints

The ability of cartilage tissue to regenerate itself is somewhat constrained due to its relative hypocellularity and avascularization. It is further impacted in horses by the significant mechanical stress and loading forces applied to the articular surfaces throughout the performance [154]. Joint problems, with osteoarthritis being the most prevalent, are one of the most frequent causes of horse athletes’ careers ending and persistent lameness [155]. Horses’ athletic performance is frequently related to the traditional remedy for musculoskeletal affections that destroy the articular cartilages, ligaments, and menisci [156]. It has been documented that intra-articular MSC therapy for cartilage, bone, and meniscal diseases in horses was beneficial *in vivo*. Bone spavin, a degenerative joint condition where standard therapy is centered on the administration of corticosteroids for reducing pain and inflammation, is the most researched and characterized locomotive system ailment in horses. The study’s findings, which involved treating 16 horses with bone spavin with autogenous intraarticular AD-MSCs, point to the beneficial and durable effects of MSC treatment. At 180 days following treatment, there was no evidence of lameness in the treated horses compared to the control group. This was corroborated by scintigraphy, which contrasted the horses’ treated tarsal joints with the control group’s still-inflamed joints and showed no evidence of an inflammatory process in the horses’ treated tarsal joints [157]. In horses with meniscal injury, MSC therapy is also quite promising. Compared to horses treated only with arthroscopy, those treated with intraarticular injections of autogenic BM-MSC had a higher percentage of horses returning to work [97]. In one study, allogeneic AD-MSCs were used to treat 80 horses with osteoarthritis, and throughout the 90-day follow-up period, a substantial decrease in lameness was seen, indicating the positive impact of allogeneic cells [98]. Similar results were shown when horses with metacarpophalangeal/metatarsophalangeal osteoarthritis were treated with allogenic UC-MSCs. However, there were no clinically meaningful improvements after 6 months. However, several investigations have shown that administering allogeneic MSCs repeatedly to osteoarthritic horses resulted in negative clinical reactions [158]. Allogeneous MSCs have been observed to cause slight to moderate topical inflammatory symptoms even after a single injection [59]. According to several studies conducted on dogs, injecting MSC into arthritic joints reduces pain and improves function in animals. The much slower course of osteoarthritis in joints treated with autogenic AD-MSCs than in joints treated with placebo revealed a considerable reduction in lameness in dogs with stifle osteoarthritis [159]. Black et al. [160] and Vilar et al. [45] observed comparable outcomes in canines with hip osteoarthritis. According to research with up to a 4-year follow-up, the effects of intra-articular administration of autogenous AD-MSCs to treat canine osteoarthritis of various joints appear to be long-lasting [161]. Utilizing allogeneic AD-MSCs has also been demonstrated to significantly enhance MSC treatment for osteoarthritis. No side effects were found in 74 dogs treated with allogeneic AD-MSCs and placebo-controlled research, and effectiveness in lowering clinical symptoms was demonstrated in contrast to the control group [162]. Another large study that had 203 dogs with serious osteoarthritis that caused significant chronic pain and lameness revealed that, 10 weeks after therapy, 90% of young dogs exhibited great recovery and 60% of older dogs showed good improvement [163]. In contrast to the control group, cartilage repair was recorded in a dog model of osteoarthritis treated with allogeneic UC-MSCs. This cartilage repair was observed as cartilage neogenesis, a diminished amount of the joint fluid, a lowered inflammatory reaction, and enhanced healing of the adjacent tissues [164]. In contrast to research done on horses, recurrent allogeneic MSC treatment has been demonstrated to be safe with only moderate, self-limiting inflammatory responses and no negative consequences even two years following intraarticular MSC injection [165]. Both autogenic and allogeneic MSC treatments for canine osteoarthritis have been examined and shown to be effective when combined with PRP or hyaluronic acid [166]. When osteoarthritis in dogs was treated with AD-MSCs and PRP, MSC therapy exhibited more potent and advantageous results [44]. ECV treatment has already been used to treat suspensory ligament injuries in a stallion, with excellent outcomes demonstrated by enhanced angiogenesis, lesion filling, and tendon flexibility [167].

#### 6.4.3. Muscles and Nerves

Spinal cord injuries (SCI), which can cause permanent disability, are among the most frequent neuromuscular injuries in both humans and animals [99]. Dogs who suffer from spinal cord damage may do so due to trauma or a herniated disc. Treatments using stem cells have been investigated and found to be effective for both illnesses. Through hemilaminectomy, autogenic BM-MSC treatment was investigated in dogs for spontaneous spinal cord damage brought on by spinal trauma. Some of the animals showed slight to moderate improvements in their locomotion, nociception, and proprioception [55,99]. In a different study, individuals with severe spinal cord injuries who received both allogeneic BM-MSCs and regular drug therapy had a statistically significant increase in their functional recovery compared to those who received only the usual medication [100]. Positive outcomes of MSC treatment were shown in dogs with acute disc herniation, much as they had been in dogs with traumatic spinal cord injuries. AD-MSCs were applied epidurally to dogs with acute paraplegia, and these dogs recovered their locomotion more quickly than dogs only receiving surgical decompression [168]. However, autogenic BM-MSC transplantation had no impact on clinical outcomes in dogs with naturally occurring degenerative intervertebral disc degeneration, and none of the three dogs presented any regenerative benefits [169]. The results of trials employing MSCs to treat traumatic spinal cord damage and disc herniation in canines did indicate some favorable outcomes, but further research is required to find a way to enhance the therapeutic advantages of MSC therapies that have already been identified. An option is a tissue engineering strategy. In one investigation, spinal cord transplantation of canine MSC-originated neural network tissue led to the progressive recovery of the motor functions of the paralyzed limb [170]. Therefore, more research and advancements are required to determine whether cell therapy and tissue bioengineering strategies are helpful for spinal cord damages, particularly in animals with spontaneous damages where the disease progression is frequently highly different from the pathologies induced in experiments.

### 6.5. Kidneys

Chronic tubulointerstitial nephritis, tubular atrophy, and interstitial fibrosis are the hallmarks of chronic kidney disease (CKD), a frequent medical problem encountered in aged cats. Currently, the only treatment that might restore renal function is kidney transplantation [171]. Therefore, stem cell-based treatments may offer less drastic therapeutic choices. Intravenous injection of stem cells is the recommended method of cell delivery since intrarenal stem cell inoculation has serious side effects and anesthesia-related dangers [101,102]. However, there were no negative effects described following IV delivery of allogeneic AD-MSCs to cats with kidney illness, and there were also no enhancements of the renal function in the short run [172,173]. However, in a work by Vidane et al. [102], cats with spontaneous CKD were regularly given IV injections of allogeneous MSCs produced from feline amniotic membranes, and a substantial improvement in kidney function was detected after the second transplantation of MSCs. Particularly, urine-specific gravity rose, and urine protein, and serum creatinine concentrations fell. The general clinical health of cats, including their eating habits and social interactions, has also significantly improved. Inconsistent findings from a few trials make it difficult to draw a firm judgment on the appropriateness of MSC treatment in cats with CKD. Thus, the employment of MSC therapy in (FCGS) has shown extremely positive results. Additional research is required to ascertain the potential impact of various variables, such as tissue source for MSCs, single or recurrent MSCs administrations, and timing of application concerning disease stage, on the outcomes of MSC treatment in cats with CKD. Further research is needed on the issue of allogeneic cell safety in cats because there has not been nearly enough studies done in this area.

### 6.6. Respiratory System

Respiratory illnesses are a common issue in veterinary medicine. Asthma, which includes several disorders including recurrent airway obstruction (RAO) and/or inflammatory airway illness, is a serious medical ailment for which there is no effective cure, particularly in horses. Moldy hay, pollen, and dusty straw are all conducive to the disease’s development. Horses frequently cough, require more effort to breathe when at rest, and are intolerant to activity. The use of corticosteroids, bronchodilators, or altering the environment can all be used to manage clinical symptoms. However, new therapeutic approaches are required to avoid the possible negative side effects of these medications. In horses with RAO, Barussi et al. [103] investigated the impact of intratracheal injections of bone marrow-derived mononuclear cells (BM-MNCs) on the progression of respiratory inflammation. In contrast to oral therapy with dexamethasone and single intratracheal delivery of autogenic cells, BM-MNCs reduced the clinical symptoms and the inflammatory reaction in horses with RAO. After cell therapy, IL-10 levels rose and were noticeably greater than in the dexamethasone-treated control group. The findings of this investigation are consistent with those of experimental studies in dogs [104] and cats that used induced respiratory circumstances [174].

### 6.7. Eye

Ophthalmology also conducts research on stem cell treatment. Some ocular conditions, such as corneal ulcers, can be treated with the present therapeutic strategies. Three cases of persistent corneal ulcers and one incidence of retinal detachment in horses that had not responded to conventional therapy were both treated with autogenous peripheral blood stem cells (PB-SCs). Cells were administered intravenously (IV), topically into the ophthalmic artery, subconjunctivally, or as an eye drop formulation. After therapy, all four animals showed considerable progress, with the epithelial surface being restored and the level of inflammation decreasing [175]. In another work, in three out of four horses, subconjunctival delivery of autogenic BM-MSC also improved immune-mediated keratitis, as evidenced by improved corneal clarity, less local neovascularization, and diminished surface abnormalities [105]. Equine recurrent uveitis (ERU) may be altered by the immunomodulatory actions of MSCs, as indicated by the reduced production of IFN- by CD4+ T cells from horses with ERU after being incubated with AD-MSCs *in vitro*. It has been demonstrated that PGE2 signaling and contact-dependent mechanisms reduce CD4+ T cell activation [176]. The outstanding outcomes exhibited by the subconjunctivally implanted allogenic AD-MSCs, including a significant reduction in the clinical manifestations of feline eosinophilic keratitis (FEK), rendered the use of MSC therapy for the treatment of these affections highly promising [106]. Moreover, keratoconjunctivitis sicca (KCS), often known as a dry eye condition, has been successfully treated in dogs with MSCs. The local allogeneic AD-MSC implantation around both lacrimal glands dramatically and sustainably decreased the clinical symptoms [177]. Likewise, the work of Sgrignoli et al. [178] showed that 6 months after recurrent local delivery of allogenic AD-MSCs into the dog conjunctival sac, exhibited a considerable reduction in the expression of KCS markers; IL-6, IL-1, TNF, and CD4. Based on the present knowledge, MSC therapy offers novel treatments for several animal eye affections, including corneal ulcers, recurrent uveitis, immune-mediated and eosinophilic keratitis, and dry keratoconjunctivitis. Though, further blinded prospective trials are required to gauge the *in vivo* impact of MSC treatment more precisely, particularly for horses with ERU. To completely comprehend the complexity and severity of certain illnesses as well as the regenerative effects of stem cell treatments used in the treatment of the eyes, ongoing scientific study is unquestionably required.

## 7. Conclusions and Future Perspectives

Research on regenerative medicine for animals is ongoing. In recent years, significant progress has been made toward producing safe and efficient stem cell treatments. Significant improvements in MSC therapy have been achieved in the treatment of various disorders, such as FCGS and IBD, as well as for wound healing. Notable effects of MSC treatments have been recorded, particularly for orthopedic problems in dogs and horses. Positive results from various studies indicate significant potential for stem cell therapy for various animal illnesses in the future. However, some obstacles still need to be resolved. The best source for MSC isolation is one of them. Most studies employed MSCs generated from adipose and bone marrow, mainly because they are accessible and simple to deal with. Therefore, it is possible that, in the future, stem cells from other organs will be shown to be more effective for treating definite disorders. Future research is also needed to determine how age and maybe even sex affect the therapeutic benefits of MSCs. There are significant differences between studies in terms of the illness progression time, cell dose, and MSC administration method. The ideal treatment methods for a certain condition are not suggested by any defined standards of the protocol. Despite claims of short viability and quick cell discharge, intravenous administration of MSCs has frequently been employed to treat a variety of animal ailments. Apoptosis-mediated immunomodulation via the immune cells is, according to some research, maybe the trigger for healing immunomodulatory mechanisms in the body, which might lengthen the effect of MSCs. Another significant problem in the realm of systemic stem cell treatments is the trapping of MSCs in the lungs following IV administration. The major substitute for IV administration of MSCs may be the systemic administration of ECVs, while different methods of administration have been taken into consideration to prevent lung entrapment. Early studies indicated that ECVs may be a promising cell-free treatment that might minimize lung entrapment and prevent potential pulmonary embolism induced by IV injection of MSCs. However, more research is required to determine the efficacy and safety of ECVs. The use of autogenic or allogeneic cells is an additional issue that has not been addressed. Although autogenic cells are safer, using them is more difficult and expensive for animal owners. Therefore, allogenic cells from healthy donors are a viable substitute, albeit there are still issues to be overcome about their immunogenicity and propensity to incite an immunological reaction in the receiver of the cells. Although veterinary regenerative medicine has made significant strides recently, this area is still in its infancy and requires a lot more effort to address many concerns before it is proven and standardized medicines can be provided to clinical cases. We are in an exciting period as more and more breakthrough regenerative medicines are developed. One may have faith that ongoing research in this field will eventually get us to a place where stem cell treatments could be a potent therapeutic option in both veterinary and human medicine for the curing of a variety of diseases that are presently incurable, rather than just a theoretical possibility.

## Data Availability

Not applicable.
